# Capturing the Dynamics of Homelessness Through Ethnography and Mobile Technology: Protocol for the Development and Testing of a Smartphone Technology–Supported Intervention

**DOI:** 10.2196/53022

**Published:** 2024-04-22

**Authors:** Marva Foster, Gemmae M Fix, Justeen Hyde, Shawn Dunlap, Thomas H Byrne, Naomi F Sugie, Randall Kuhn, Sonya Gabrielian, Jill S Roncarati, Shibei Zhao, D Keith McInnes

**Affiliations:** 1 Center for Healthcare Organization and Implementation Research VA Boston Healthcare System Boston, MA United States; 2 Department of General Internal Medicine Boston University Chobanian & Avedisian School of Medicine Boston, MA United States; 3 Center for Healthcare Organization and Implementation Research VA Bedford Healthcare System Bedford, MA United States; 4 Department of Social Welfare Policy Boston University School of Social Work Boston, MA United States; 5 Department of Criminology, Law and Society University of California Irvine Irvine, CA United States; 6 Department of Community Health Services Fielding School of Public Health University of California Los Angeles Los Angeles, CA United States; 7 Department of Mental Health VA Greater Los Angeles Los Angeles, CA United States; 8 Department of Psychiatry and Biobehavioral Sciences David Geffen School of Medicine University of California Los Angeles Los Angeles, CA United States; 9 Department of Health Policy and Management Harvard TH Chan School of Public Health Boston, MA United States; 10 Boston Health Care for the Homeless Program Boston, MA United States; 11 Department of Health Law, Policy and Management Boston University School of Public Health Boston, MA United States

**Keywords:** ethnography, homelessness, housing transitions, longitudinal data, military, mobile technology, smartphone, social support, veterans

## Abstract

**Background:**

US military veterans who have experienced homelessness often have high rates of housing transition. Disruptions caused by these transitions likely exacerbate this population’s health problems and interfere with access to care and treatment engagement. Individuals experiencing homelessness increasingly use smartphones, contributing to improved access to medical and social services. Few studies have used smartphones as a data collection tool to systematically collect information about the daily life events that precede and contribute to housing transitions, in-the-moment emotions, behaviors, geographic movements, and perceived social support.

**Objective:**

The study aims to develop and test a smartphone app to collect longitudinal data from veterans experiencing homelessness (VEH) and to evaluate the feasibility and acceptability of using the app in a population that is unstably housed or homeless.

**Methods:**

This study’s design had 3 phases. Phase 1 used ethnographic methods to capture detailed data on day-to-day lived experiences of up to 30 VEH on topics such as housing stability, health, and health behaviors. Phase 2 involved focus groups and usability testing to develop and refine mobile phone data collection methods. Phase 3 piloted the smartphone mobile data collection with 30 VEH. We included mobile ethnography, real-time surveys through an app, and the collection of GPS data in phase 3.

**Results:**

The project was launched in June 2020, and at this point, some data collection and analysis for phases 1 and 2 are complete. This project is currently in progress.

**Conclusions:**

This multiphase study will provide rich data on the context and immediate events leading to housing transitions among VEH. This study will ensure the development of a smartphone app that will match the actual needs of VEH by involving them in the design process from the beginning. Finally, this study will offer important insights into how best to develop a smartphone app that can help intervene among VEH to reduce housing transitions.

**International Registered Report Identifier (IRRID):**

DERR1-10.2196/53022

## Introduction

### Background and Rationale

US military veterans are at greater risk of homelessness compared to their civilian counterparts [1,2]. Those experiencing homelessness are at increased risk of premature mortality, with a 2-decade–shorter life expectancy than the general population [3]. They experience an elevated burden of mental illness, substance use disorder, hypertension, and cardiovascular disease [4-6] as well as infectious diseases such as HIV or AIDS, tuberculosis, and hepatitis C [7]. Although homelessness is often episodic, it can occur over a long period of time for some. Commonly, those experiencing homelessness transition between different types of accommodations: homeless shelters, doubled-up with family or friends, or staying in places not meant for human habitation (eg, encampments, vehicles, and abandoned buildings) [1]. These frequent housing transitions likely exacerbate poor health, social well-being, and economic vulnerability [8,9]. There is a major gap in detailed, contextual knowledge of these transitions, such as information about in-the-moment emotions, behaviors, geographic movements, and perceived social support.

Having a greater understanding of these in-the-moment experiences may help inform efforts to better identify people who are at risk of an unexpected or unwanted housing transition and intervene to prevent a homeless episode. Without real-time information, it is difficult to understand the point-in-time contextual nuances of an individual’s housing transition and provide support to mitigate these transitions. Previous work, whether quantitative or ethnographic, has been limited by the methodological difficulty of retrospectively gathering this type of contextual data about distinct points in time in the past [10,11]. Studies show that perceptions of emotions and behaviors are unreliable when gathered retrospectively [12,13]. Another factor that has limited understanding of the nuances of transition among veterans experiencing homelessness (VEH) is their lack of trust in people generally and in certain entities in particular (eg, “government”) [14]. Previous research indicates that smartphones are already being widely used by homeless populations, and those populations view smartphones as a trusted source to communicate and receive information [15,16]. Smartphone technology shows promise for enhancing our understanding of short-term precipitants and helping us gain a better understanding of the in-the-moment experiences that lead to housing transitions.

Smartphone apps support the collection of real-time data, which involves the use of ecological momentary assessment (EMA). EMA allows researchers to measure participants’ real-world emotions and behaviors in real-time in their natural environments [17]. Mood has been identified in numerous studies as being predictive of activity; it interacts with social support [18], is an indicator of daily stressors [19], and acts as a mediator of a variety of outcomes [20]. Additionally, technology such as GPS, which can be collected through smartphones, can provide accompanying information on daily changes in geographic movement. When analyzed with other data, this may provide insight on potential factors that precipitate a housing transition. Yet few studies have used smartphone apps to gather detailed, contextual knowledge on housing transitions among VEH. Notably, smartphone ownership among persons experiencing homelessness is growing and almost equivalent to that of the general population [21,22].

Thus, this study is among the first to design and test the feasibility and acceptability of a smartphone app that uses real-time surveys and GPS to collect longitudinal data from a population that is unstably housed. This protocol paper details the specific phases and methods used to prepare for and conduct a longitudinal smartphone data collection study with a sample of veterans.

### Objective

We aim to develop and test a smartphone app to collect longitudinal data from VEH and to evaluate the feasibility and acceptability of using the app in a population that is unstably housed or homeless.

## Methods

### Participating Institutions

The US Department of Veterans Affairs (VA) Center for Healthcare Organization and Implementation Research (CHOIR) leads the study. CHOIR invited university partners to participate to provide the types of additional expertise needed for this initiative. Joining the team are members of Boston University’s Software and Application Innovation Lab (SAIL). SAIL is a professional research, software engineering, and consulting lab that acts as both a driver and a collaborative partner for computational and data-oriented research efforts. Also joining are RK, a demographer from the University of California Los Angeles, and NFS, a sociologist from the University of California Irvine. RK brings expertise in defining study populations, including extensive experience studying and conducting interventions for persons experiencing homelessness, and NFS has a depth of expertise in developing smartphone data collection tools for vulnerable populations.

### Design

**Figure 1:**
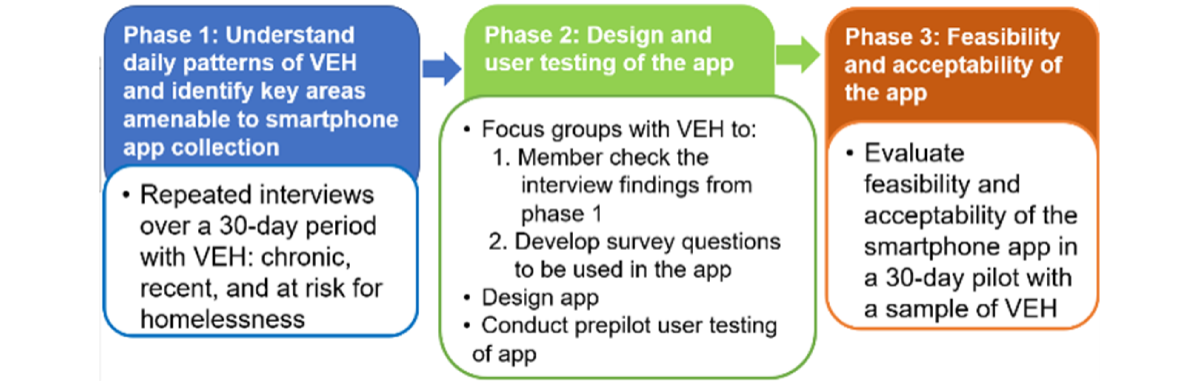
Project design showing the 3 phases of the project. VEH: veterans experiencing homelessness.

This multistage study involves identifying the needs of VEH, designing an app, and pilot-testing it (Figure 1). Phase 1 involves repeated interviews over a 30-day period to understand app use and contexts of app use among persons at risk for or experiencing either chronic or recent homelessness. Phase 2 involves focus group discussions with VEH to members check the interview findings from phase 1 and develop survey questions to be used in the designed app. Additionally, user testing methods will be used to refine questions as needed. Lastly, in phase 3, we will test the feasibility and acceptability of the developed app with a sample of VEH.

### Study Settings

The study is being conducted at 2 locations in the northeastern region of the United States to achieve diversity in the participant samples. Site 1 is one of the VA health care system’s 172 medical centers, located about 15 miles north of a major city. It is one of the largest providers of services to VEH and provides a range of health and social services to veterans living in suburban and rural areas. This site is a multiservice residential program serving veterans who are experiencing homelessness or at risk for homelessness. Site 2 is located in the center of a dense urban area. This site is a multiservice community-based residential program serving veterans who are experiencing homelessness and at risk for homelessness.

### Phase 1: Understand Daily Patterns of VEH and Identify Key Areas Amenable to Smartphone App Collection

#### Sampling of Participants and Recruitment

Using a convenience sampling strategy, we will recruit participants from 3 locations with a range of experiences with homelessness, including chronic homelessness, a recent onset of homelessness, or being at-risk for homelessness. For the purposes of this study, we define chronic homelessness as having a pattern of multiple episodes (or a single long episode) of homelessness over an extended period (ie, a year or more). We define recent onset as newly homeless, with the period of homelessness having begun in the last 6 months but not having a history of being chronically homeless. Lastly, we define being at risk as being at imminent risk of losing one’s housing [23]. Recruitment will begin with the study team hosting informational sessions, followed by sharing and posting flyers at the residential programs at sites 1 and 2. Those who express interest will be approached by study team members during informational sessions or later contacted by telephone. They will then be screened for their current homeless experience (chronic, recent-onset, and at-risk) to confirm that they meet eligibility criteria.

#### Procedures

A 30-day focused ethnographic data collection design will be used to understand the daily patterns of VEH and identify key areas amenable to smartphone app collection. Individuals will be interviewed multiple times over a 30-day period. This time frame will be consistent with the one planned for the phase 3 pilot study of smartphone-enabled data collection and will offer proof of the concept of engagement in this later work. The focused ethnographic approach will include four different types of interviews. Interview types will include (1) a baseline, historical interview (with structured and open-ended components) that will last between 60 and 90 minutes; (2) long interviews that will focus on a “special topic” (perceptions of health, social services, and technology, including the use of smartphones) of interest to the study and will last approximately 60 minutes; (3) a general short interview, twice a week, that will last approximately 15 minutes; and (4) a final follow-up interview to obtain feedback on participation in the multiweek study. Textbox 1 shows an overview of the interview schedule, and Textbox 2 shows a description of interview topics.

Both the long and short interviews will provide an opportunity to ask VEH their opinions about the most important things to ask on a regular basis that might influence stability in housing, how to ask about sensitive topics like substance use, and about potential concerns with answering questions through a smartphone app.

**Phase 1 30-day illustrative interview schedule.**

**Weeks and interview schedule**

- Week 1: baseline interview 1, short interview 2, short interview 3, and long interview 4
- Week 2: short interview 5, short interview 6, and long interview 7
- Week 3: short interview 8, short interview 9, and long interview 10
- Week 4: short interview 11, short interview 12, and final interview 13

**Interview types and the structured and unstructured ethnographic data collected over the course of the phase 1 30-day interviews.**

**Interview types and descriptions**

- Baseline interview: This interview is designed to:
  Gain a deeper understanding of participants’ housing history using a modified Residential Time-Line Follow-Back Inventory [24].
  Collect information on key social relationships, military experiences, and employment opportunities that may have influenced the life course of veterans experiencing homelessness.
  Collect and record demographic information on a short survey form.
- Long qualitative interviews: longer “special topic” interviews to gain insights into 3 different topics
  Physical and mental health (eg, perception of overall health, experience with physical and mental health conditions and the extent to which they impact everyday life, and perceptions of the impact of health conditions on housing stability) and its impact on housing stability.
  Access to and use of social services (eg, places participants go for financial, logistical, or social support to meet needs, facilitators, and barriers to accessing different types of support, and perceptions of how support influences housing stability).
  Use of technology (eg, types and uses of phones, including common apps and phone features).
- Short qualitative interviews: interviews will occur 2-3 times per week, last approximately 15 minutes, and happen between the longer special topic interviews. These interviews will explore participants’ feelings, where they slept the night before, and any changes in their lives since the last conversation.
- Final interview: a 60-minute interview to obtain feedback from participants on the experience of engaging with a research team over a prolonged period.

#### Analysis Phase 1

##### Quantitative Data Analyses

Demographic characteristics like recent life experiences, homelessness episodes, overall health and health conditions, and social service use of participants will be entered into an Excel (Microsoft Corporation) spreadsheet. We will use descriptive statistics to summarize the characteristics of these participants.

##### Qualitative Data Analyses

All interviews will be professionally transcribed verbatim. Data will be analyzed using a Rapid Assessment, Response, and Evaluation approach [25], which uses a multidisciplinary team to collect different types of data that can be synthesized and analyzed iteratively and efficiently to generate an understanding of critical health and public health issues. Each participant in phase 1 will have a portfolio of data from their multiweek study period. Analysis will entail attention to both the cumulative story and key changes that might impact housing transitions or health. Once the transcript of each interview is available, a standardized template will be created to systematically summarize data with attention to transitions in housing and health for each participant. The summarized information will be examined to identify patterns, common concepts, and emerging ideas about current events and experiences that influence transitions in housing and health. For each participant, the lead interviewer will compile and analyze the portfolio of data. They will create a summary template that systematically synthesizes the data across the 4 weeks of data collection. This summary template will contain key fields such as transitions in housing and health for each participant, as well as key events such as fluctuations in mood and disposition, social networks, and experience with health and social services. After completion, a second team member, not involved in that participant’s data collection, will review the summary template. The team will discuss the data until consensus is reached. This process will ensure consistency, clarity, and rigor. Following this process, team members will be designated to draft questions aligned with transitions in housing, health, and key events. These members will be instructed to first look for validated questions that may be appropriate. If none exist, they will draft questions for group review. Through a series of meetings, the full team will review all drafted questions and make decisions about which ones to include, exclude, and modify for a smartphone app format. This information will be used to inform phase 2 data collection and analysis.

### Phase 2: Design and User Testing of the App

#### Sampling of Participants and Recruitment

Phase 2 will use the same recruitment and consenting strategy as phase 1. Phase 2 focus group participants will be recruited from site 2.

#### Procedures

##### Focus Groups

Focus groups will take place at a nearby VA hospital and on-site at the residential program. These discussions will provide an opportunity to build on the phase 1 findings and further explore how to recruit, enroll, and retain VEH in a future, app-based study planned in the next phase. The focus group participants will be asked for their general perceptions of taking part in a research study that uses a smartphone app for data collection and recommendations regarding how to (1) introduce and explain a study that uses the collection of data through a smartphone app, (2) explain the ways in which privacy and confidentiality will be maintained, (3) ask about potentially sensitive topics, such as substance use and relationship conflict, and (4) assess capacity to participate using a smartphone app. Participants will also be provided a brief questionnaire to obtain specific information about their access to and use of technology generally, and specifically smartphones.

We will also inquire about what types of information participants would be willing to provide researchers through their mobile phones, the acceptability of having GPS locations tracked, and any locations where they would not want GPS. We will show the focus group participants a mockup data map of an individual’s GPS generated locations and movements to facilitate discussion. We will also inquire about other data collection methods, such as passively collected call logs. We will share with participants mockups of what call log data would look like to researcher team members. We will also emphasize that there will be encryption built into the software to address concerns about privacy. Feedback from the focus groups will be used to adjust the prototype of the smartphone app and guide revisions to phase 3 protocols. Focus group discussions will last approximately 60 minutes and will be facilitated by one of the anthropologists on the study team.

##### Development and Usability Testing

Smartphone app development will be informed by data from phase 1 ethnographic and phase 2 focus group findings. Usability testing will involve 5 VEH participants reviewing app mockups. Participants will be asked to provide feedback about the home screen, survey screen layouts, survey response options, and settings screen. Usability testing will occur approximately 2-3 months after the focus groups, when the mobile app has reached functionality. SAIL team members will provide mobile phones preloaded with the app. A member of the study team with qualitative expertise will record notes in a data collection template about overall design, ease of use, and specific wording and phrases used in the app during usability testing. Participants will use a smartphone to complete a defined set of tasks, such as turning on the phone, turning GPS off and on, responding to EMA and survey questions, etc. We will follow usability protocols and assessments, including time to complete tasks, participant error rates, software errors, etc. The team member will also ask participants about the burden of answering questions and about issues of privacy and trust. The SAIL development team will incorporate lessons from focus groups and usability assessment into the final version of the smartphone app for the phase 3 pilot. Usability assessments will be conducted by research staff who have been trained in usability assessment procedures by the SAIL mobile development group.

#### Analysis Phase 2

##### Phase 2 Focus Groups

The phase 2 focus groups and mobile app design data will include (1) the use of focus groups to gather information about the frequency of data collection, privacy issues, and incentives, and (2) the use of both phase 1 ethnographic data and phase 2 focus group data to develop a smartphone app and perform app usability testing.

##### Qualitative Data Analyses

All focus groups will be professionally transcribed verbatim. Thematic analysis [26] will be used by the focus group leads to analyze the data, which will be shared with the full team for an analytic discussion. Transcripts will later be combined with the phase 1 data for a global discussion of implications for the phase 3 pilot.

### Phase 3: Feasibility and Acceptability Pilot of the App

#### Sampling of Participants and Recruitment

Phase 3 will use the same recruitment and consenting strategies as the previous phases. Phase 3 participants will also be recruited from sites 1 and 2.

#### Procedures

##### Baseline Interview and Demographics

At enrollment in phase 3, study team members will conduct semistructured qualitative interviews to gather structured data on demographics, recent life experiences, homelessness episodes, overall health and well-being, impairments, supportive relationships, and technology use. During the interview period, participants will be guided on how to download the app and will be provided with an orientation on how to use the app. The baseline interview will last about an hour.

##### 30-day Smartphone App Data Collection

We will recruit up to 30 participants who will use the app to daily answer survey questions related to housing transitions, health service usage, medication adherence, emotional states, and social relationships [27,28]. About 2 days a week (Wednesday and Saturday evening), participants will receive a longer app survey with additional questions about health care, substance use, and the ongoing quality of specific social relationships. The questions on substance use and social relationship quality will be based on data collected during the baseline survey and qualitative interview. We will also collect information on veterans’ activity space (the local areas within which people move or travel over the course of their daily activities) and proximity to health or social services by using GPS [29]. GPS data will primarily be used to characterize the overall extent and variability of the activity space.

##### Follow-Up Interview

At the end of the 30-day pilot, a final follow-up interview will be conducted, using both structured and qualitative components. The interview will be conducted to document participant experiences and perceptions about the app data collection, including issues relating to trust, data security, maintaining phones, and the usability of EMA and mobile surveys. Participants will be allowed to view their survey and GPS data to offer insight into specific ambiguous survey responses and validate the general accuracy of the data set. This information will be used in a later analysis.

#### Analysis Phase 3

The phase 3 feasibility and acceptability pilot of mobile phone data will include 2 interviews (baseline and follow-up), as well as the mobile survey, GPS, and EMA data.

##### Qualitative Data Analyses

Baseline and final interview data will be organized and analyzed using the processes described in phase 1. The coding will closely follow the topics covered in the interview guide around performance and effort expectancy, social norms, facilitating factors, and trust.

##### Quantitative Data Analyses

First, we will use descriptive statistics to summarize the structured items collected from the baseline interviews, including information about participants’ demographic characteristics, recent life experiences, homelessness episodes, overall health and health conditions, and social service use. Second, we will use sequence analysis to identify distinct patterns of housing transitions. Third, we will perform convergent integration of the qualitative and quantitative data to assess how contextual characteristics, including mood, activity space size, social support, medication adherence, and usage of health services, are related to housing transition patterns. Fourth, we will use mixed effects regression models to assess how daily changes in contextual characteristics are associated with daily experiences of housing stability (and major health events).

### Ethical Considerations

Ethical approval was given by the VA Bedford Institutional Review Board (1626836-16), in accordance with VA guidelines. Written informed consent will be sought from all participants. Phase 1 participants will be provided written informed consent and will receive up to US $185 reimbursement over the course of the study (US $25 for the baseline, follow-up, and long interviews and US $10 for each brief interview). Phase 2 participants will receive US $25 upon completion of their focus group. Phase 3 participants will be offered US $30 for the baseline and follow-up interviews and US $5 per survey completed in the app for a possible total compensation of US $340. Study ID numbers will be assigned to participants to ensure confidentiality. We sought additional protections and obtained a federal Certificate of Confidentiality from the US National Institutes of Health because of the sensitive nature of the study discussions. Certificates of confidentiality protect the privacy of research subjects by prohibiting the disclosure of identifiable, sensitive research information to anyone not connected to the research except when the subject consents or in a few other specific situations. It is not possible to create a minimal data set with this qualitative data.

## Results

The project was launched in June 2020, and as of December 2022, we had enrolled 10 participants for phase 1 and 9 participants for phase 2. Data collection and analysis for phases 1 and 2 are complete. This project is currently in progress.

## Discussion

### Principal Results

VEH are extremely vulnerable to experiencing housing transitions compared to their civilian counterparts [1,2]. A significant gap remains in research approaches to understanding the short-term precipitants and influences upon housing transitions. The use of smartphone apps can help fill the significant gaps in our knowledge, including, (1) the sequence of events leading up to and immediately after housing-related transitions; (2) information about the housing status of veterans not included in administrative systems, such as those who are living double-up or are street homeless; and (3) potential “early warning” signals gleaned from near-real–time reports of mood, activities, social support, and activity spaces (constructed from passively collected GPS data) that may presage increasing housing instability, a homelessness episode, or a major health event. This protocol paper details the specific phases and methods that will be used to develop and test a smartphone app to collect longitudinal data from VEH and to evaluate the feasibility and acceptability of using the app in a population that is unstably housed or homeless. This multiphased research will provide contextual knowledge on how in-the-moment experiences precipitate housing transitions from the perspective of VEH.

Furthermore, engaging participants from a range of housing situations—street homeless, transitional housing, residential treatment programs, and shelters—in the development of the smartphone app will allow us to capture what VEH thinks is significant to include in the app, thus enhancing its potential uptake [30,31]. To our knowledge, this will be the first test of active and passive smartphone-enabled data collection applied to the study of homelessness among veterans. At a minimum, the information gained from this project will lay the groundwork for enhancements to clinical programs that incorporate smartphone data for early intervention or use smartphone tools for 2-way communication between patients and clinical teams to improve care access and engagement for vulnerable populations.

### Limitations

While our design methods during phases 1 and 2 will offer rich and nuanced insights to guide the content and design of the smartphone data collection app, there are a few limitations worth noting. First, there are usually difficulties in contacting and recruiting certain VEH, which could bias this study because we may miss the most vulnerable and extreme cases. Thus, we will work closely with our staff at sites 1 and 2 to reduce selection bias. Second, VEH tends to report a lack of trust due to poor previous experiences with health care providers and mistrust of government institutions. We will try to establish a relationship of trust between the interviewer and VEH participants to obtain the most reliable data possible. Finally, although this will be an evaluation of VEH, our results may be applicable to non-VEH populations, contexts, and activities.

### Conclusions

This multiphased study will provide rich data on the context and immediate events leading to housing transitions among VEH. This study will ensure the development of a smartphone app that will match the actual needs of VEH by involving them in the design process from the beginning. Finally, this study will offer important insights on how best to develop a smartphone app that can help intervene among VEH to reduce housing transitions.

## Abbreviations

CHOIR: Center for Healthcare Organization and Implementation Research
EMA: ecological momentary assessment
SAIL: Software & Application Innovation Lab
VA: Veterans Affairs
VEH: veterans experiencing homelessness

## Data Availability

The underlying data for this study consists of in-depth, qualitative interviews with veterans experiencing homelessness. The data sets generated or analyzed during this study are not publicly available due to participant privacy but may be available from the corresponding author.
